# Reducing undiagnosed HIV infection among adolescents in sub-Saharan Africa: Provider-initiated and opt-out testing are not enough

**DOI:** 10.1371/journal.pmed.1002361

**Published:** 2017-07-25

**Authors:** Marguerita Lightfoot, Megan Dunbar, Sheri D. Weiser

**Affiliations:** 1 Division of Prevention Science, Department of Medicine, University of California San Francisco, San Francisco, California, United States of America; 2 Pangaea Zimbabwe AIDS Trust, Harare, Zimbabwe; 3 Division of HIV, Infectious Disease and Global Medicine, Department of Medicine, University of California San Francisco, San Francisco, California, United States of America

## Abstract

Community efforts and peer support programs are needed in addition to provider-initiated and opt-out HIV testing in adolescents, Sheri Weiser and colleagues discuss.

While identifying individuals with HIV infection and linking them to subsequent antiretroviral (ARV) treatment can be effective in reducing HIV transmission at the population level, these efforts are hampered by the lack of HIV testing uptake among older children and adolescents. The prevalence of undiagnosed HIV infection is significantly higher among older children and adolescents than adults [1], particularly in sub-Saharan Africa, where only 13% of girls and 9% of boys have been tested for HIV and know their results [2]. This leads to significant morbidity and mortality among children and adolescents, who are often not diagnosed until the onset of AIDS. Furthermore, these undiagnosed infections continue to drive the epidemic, particularly as adolescents become sexually active. As such, the World Health Organization has prioritized older children and adolescents as a key population for HIV testing [3]. The paper by Simms and colleagues [4] appearing in this week’s *PLOS Medicine* examines the impact of an important strategy, provider-initiated testing and counseling (PITC) among older children and adolescents in Zimbabwe.

## The limitations of PITC

A recent systematic review concluded that PITC is the most commonly used strategy for HIV testing to identify undiagnosed children and adolescents, likely because of its seeming ease to implement and its documented high uptake and yield in previous studies [5]. The review highlighted that this strategy is more likely to identify those adolescents already showing symptoms of HIV and who may therefore have more advanced disease, which prompts their provider to offer an HIV test [5]. However, Simms and colleagues hypothesized that by offering PITC over a 2-year period in primary care settings through an opt-out testing approach, most older children and adolescents living with undiagnosed HIV would likely present for primary care at some point, and thus, this approach would identify a higher proportion of undiagnosed nonsymptomatic HIV infection than is typical of PITC.

The authors found that uptake of testing when offered by providers at primary care clinics was relatively high (79.3%), and based on the household survey in areas served by those clinics, they found that 37.7% of HIV-infected patients were previously undiagnosed. Extrapolating from their sample to the target population, they estimate that PITC identified between 18% and 42% of previously undiagnosed children and adolescents in the community. Over one-quarter of older children or adolescents with unknown HIV status who presented to care did not have a parent or suitable guardian with them and hence were not eligible for testing. Additionally, even among those with a suitable guardian, and with additional staff and test kits, 20.7% of patients did not receive an HIV test. These findings echo the limitations of this strategy, including the selective offering of HIV testing by providers even when presented as opt-out, lack of guidance for consenting adolescents and their guardians in the clinical setting, and logistical constraints. While this study supports PITC as a useful model, it highlights the need to implement PITC in combination with additional testing and outreach strategies, particularly at the community level, to effectively reach undiagnosed older children and adolescents.

## The challenges of HIV testing for adolescents

Implementers planning HIV testing and counseling strategies for adolescents must consider a number of critical issues. Adolescents have the lowest rate of primary care use among any age group [6]. Many adolescents with asymptomatic HIV or sexually transmitted infection (STI)-related symptoms do not seek treatment services [5], and those who do access the healthcare system may not be screened for HIV even when PITC is targeted for them [7]. Compounding these issues in sub-Saharan Africa are the numerous barriers experienced by adolescents in accessing medical care, particularly the lack of adolescent-friendly services available during clinic hours that do not conflict with school attendance, adolescents’ lack of knowledge about HIV/STIs and available services, costs of services or transport to clinics, and HIV-related stigma [8]. When children and adolescents do access services and when PITC is offered, they often encounter providers who are not trained in or responsive to developmental issues or who can be judgmental in their approach [8]. In addition, the potential impact of parents, caregivers, and families on HIV testing in sub-Saharan Africa cannot be underestimated. The identification of a child or adolescent’s HIV status can represent potential exposure of the parent’s positive HIV status. Also, parents’ fear of HIV stigma and likely adverse consequences, worry about their child or adolescent’s emotional reaction, and the desire to protect their son/daughter’s childhood can result in a lack of parental support and consent for HIV testing. Further, families often perceive adolescents as being at lower risk for HIV, and cultural taboos can undermine conversations with adolescents about sex and HIV risk. The lack of parental or family support for HIV testing is a particularly salient challenge, as allowing adolescents to consent for sexual and reproductive health services without parental knowledge is not universal across sub-Saharan Africa.

## Developmentally appropriate approaches for HIV testing

A combination of strategies to reach older children and adolescents and address their developmental and other unique needs is necessary to reduce the prevalence of undiagnosed HIV infection in this population. As Simms and colleagues note, innovative and effective community-based interventions to increase access and uptake of HIV testing among children and adolescents are warranted. One potentially cost-effective strategy is family-based testing, in which index cases of parents or family members living with HIV provide entry to offering HIV testing within a household. This case-finding strategy has been successfully employed in some STI [9] and tuberculosis [10] screening programs. Utilizing multidisease community health campaigns that incorporate HIV testing is another important strategy that has shown promise in reaching undiagnosed adolescents in Uganda and Kenya [11]. In addition, efforts to overcome the potential challenges posed by school-based testing could be important. Issues of parental consent, confidentiality, and linkage/follow-up to care for students who test positive can be addressed through integration with school health programs, educating school administrators and teachers about the age of legal consent for HIV testing (where appropriate), and partnerships with nongovernmental organizations. Peer-driven models in which adolescents mobilize their social networks for testing may be particularly appropriate. Peers have the unique advantage of increased credibility in delivering messages, the ability to speak from their own experiences, and the potential of reaching those individuals in their social and sexual networks who are out of touch with healthcare settings and have been successful at effectively promoting behavior change [12]. The Africaid/Zvandari program, which uses peers to support adherence to HIV treatment and retention in care for adolescent and young adults living with HIV, has been cited as such a model in the WHO’s adolescent HIV testing guidelines [3]. Another important strategy is distribution of HIV self-test kits in home and community settings, which has resulted in high acceptance, uptake, coverage, and yield among adolescents [13]. Attention to adolescent-specific barriers to HIV testing could also guide efforts to strengthen PITC—for example, expanding clinic hours to after school and weekends, enacting policies that allow adolescents to consent for testing, or providing training to providers on adolescent development. This list of potential strategies to reduce unidentified infection in older children and adolescents is not exhaustive, and the empirical evidence is mixed. Thus, additional research that examines the efficacy and effectiveness of focused strategies and combined approaches is critical.

In summary, as the article by Simms and colleagues illustrates, PITC can play an important role in increasing uptake of testing among older children and adolescents but needs to better address barriers to testing in this population and is likely not sufficient on its own. Community-, home-, and school-based approaches that recognize the unique needs of adolescents will also be required in order to adequately address the problem of undiagnosed HIV infection among older children and adolescents.

## Abbreviations

ARV: antiretroviral
PITC: provider-initiated testing and counseling
STI: sexually transmitted infection
